# Cultural hybridity and body image formation: exploring the experiences of Wushu male practitioners at the Siberian Chinese Martial Arts Center

**DOI:** 10.3389/fpsyg.2024.1435647

**Published:** 2024-10-23

**Authors:** Ekaterina A. Santanna, Benqian Li

**Affiliations:** School of Media and Communication, Shanghai Jiao Tong University, Shanghai, China

**Keywords:** cultural hybridity, body image formation, Wushu male practitioners, thematic analysis, narrative interviews

## Abstract

This study explores the formation of a hybrid body image among white cisgender males practicing Wushu at the Siberian Chinese Martial Arts Center. Grounded in Social Identity Theory and the concept of bicultural identity, the research employs narrative interviews with 12 participants. Thematic analysis revealed three main themes: the creation of a hybrid identity blending Chinese martial arts traditions with Russian cultural backgrounds, conceptualized as judanren; the challenges associated with this identity in both Russian and Chinese contexts; and the role of Wushu uniforms in reconciling these cultural contradictions, serving as a “second skin” for practitioners. The findings contribute to a more inclusive understanding of body image and identity formation in a multicultural, non-Western context.

## Introduction

The practice of martial arts has a profound impact not only on the physical condition of practitioners but also on their mental well-being (Bu et al., 2010). As mind–body practices, martial arts have been found to enhance self-confidence and shape self-body image (Pedrini and Jennings, 2021). Wushu, or Chinese martial arts, not only offers unique physical and mental exercises but also serves as a manifestation of Chinese traditional culture (Li and Dai, 2021). Rooted in ancient philosophy, Wushu embodies a unique understanding of the relationships between humans and nature, humans and others, and the connection between the mind and body (Theeboom and De Knop, 1997; Park and Bairner, 2023; Tzeng et al., 2023). Despite its Chinese culture orientation, Wushu has gained tremendous popularity worldwide (Han et al., 2023; Xue et al., 2023).

While there is a substantial body of literature on the health and mental health benefits of Wushu as a physical exercise (Wąsik and Wójcik, 2017; Moore et al., 2020), there is a dearth of studies that analyze the impact of Chinese traditional cultural elements within this martial art on participants’ perceptions of the practice and themselves.

This study addresses this gap by examining practitioners in Russia, particularly in Siberia.

In recent decades, Russia has increasingly oriented itself towards the East (Rodkiewicz, 2014), characterized by strengthened cooperation with China and a strategic distancing from the West, particularly following the invasion of Ukraine (Huang and Wang, 2024). This cultural shift is evident in various governmental initiatives that actively promote Chinese traditional culture through festivals, fairs, and other cultural events (Matveevskaya and Ren, 2023). Additionally, the growing popularity of Chinese cultural media, such as TV series, has been driven by online communities (Zhou, 2021), resulting in heightened interest among Russians in learning Chinese, joining Chinese martial arts centers, and participating in traditional Chinese painting studios.

Although Siberia does not share a direct border with China, it holds significant cultural importance due to its historical connections and the presence of diverse ethnic groups, including the Buryats, Yakuts, Tuvinians, Khakas, and Altaians. These communities contribute to a culturally rich landscape that resonates with elements of Chinese traditional culture.

Through this study, the experiences of Siberian Wushu practitioners will be explored, particularly regarding how their practice influences their body image perceptions.

## Theoretical framework

The theoretical framework employed in this study integrates two complementary perspectives: Social Identity Theory (Tajfel and Turner, 1985) and the concept of bicultural identity within Acculturation Theory (Berry, 1980). This framework provides insights into how body image is shaped by group dynamics and cultural integration. Social Identity Theory (Tajfel and Turner, 1985) posits that individuals derive significant aspects of their identity from their affiliations with social groups. This study applies this theory to examine the effects of participation in Wushu groups on practitioners’ self-perceptions, elucidating the dynamics of body image within the martial arts context.

Furthermore, the concept of bicultural identity, as defined by Berry (1980, 1997), refers to individuals engaging with two distinct cultures, reflecting an integration strategy of acculturation. Benito-Martinez et al. (2002) elaborate on this by indicating that individuals synthesize elements from both their heritage and receiving cultures, resulting in a unique bicultural identity. This study specifically investigates how Wushu practitioners navigate their dual cultural affiliations—Chinese traditional practices and their Russian environment—and how these affiliations influence their body image perceptions.

## Method

As noted by Landor et al. (2024), previous research on body image has predominantly focused on Euro-American samples and primarily employed quantitative methodologies. To expand this scope, the present study aims to explore body image perspectives among a unique group of Wushu practitioners in Siberia through a qualitative approach. Jennings (2010) advocates for the use of qualitative methods, particularly life history interviews, as effective tools for understanding the transformations individuals undergo through long-term practice of traditional Chinese martial arts.

In line with this approach, narrative interviews were selected as the primary method for data collection due to their capacity to capture in-depth, contextually rich, and participant-centered accounts that reveal individual experiences and cultural meanings (Holmqvist and Frisén, 2010). These interviews addressed a broad range of questions that aligned with the theoretical framework, covering general experiences in Wushu practice with fellow practitioners, as well as specific inquiries about self-perception and body image during practice and in everyday life.

### Context of study

The Siberian Chinese Martial Arts Center is an independent facility founded by Russian Wushu practitioners who have studied in China. These practitioners are proficient in Chinese and, after establishing the center in Russia, maintain connections with their masters to organize joint events in both Russia and China. The center emphasizes that martial arts practice extends beyond physical exercises, encompassing the philosophy of Wushu and Chan Buddhism. The goal is to strengthen not only the body but also to achieve inner peace and harmony with oneself and the world. Regular discussions and question-and-answer sessions about all aspects of practice often follow classes in the kwoon rooms.

At the center, individuals can also engage in various activities, including Wushu and Qigong classes, meditation sessions, Chinese language lessons, and the exploration of Chinese calligraphy and traditional Guohua painting. Additionally, there is a recreation zone where visitors can enjoy Chinese tea, listen to traditional Chinese music, or play Xiangqi (Chinese chess) after classes. The center also serves as a venue for celebrating Chinese holidays together. To deepen their understanding of Chinese culture, participants can take part in master classes on Chinese cuisine, traditional knotting, or shadow theater.

### Participants

All participants were recruited through advertising and researcher invitations during training sessions at the Siberian Chinese Martial Arts Center. Jennings (2010) notes that “four years is a typical period for a student to progress from a beginner to senior student and possibly even assistant instructor status within a school” (p. 26). This aligns with the program structure at the center, where students spend their first year learning basic concepts and foundational elements before mastering the first taolu complexes, which include various movements, transitions, punches, and stances.

Although students’ progress at varying speeds and possess different abilities, deep immersion in Wushu practice generally does not commence until after at least one year of practice. As a result, one year of practice was established as the minimum criterion for participation in this study. However, while individuals with approximately one year of practice were eligible, only those with a minimum of two years of training chose to participate, reflecting a greater commitment to their training and readiness to engage in the research.

Moreover, despite invitations being extended to participants of all genders in the Wushu classes, only male participants opted to take part in the study. Consequently, the sample comprised twelve cisgender males who voluntarily agreed to participate. All participants were Russian citizens and identified as white. Their ages ranged from 27 to 55, with a mean age of 36.2 (SD = 8.2). The participants’ experience in Wushu varied from 2 to 20 years, with one individual practicing for 20 years, two individuals for 10 years, and the remaining participants for periods between 2 to 5 years.

In terms of education, ten out of the twelve participants had a higher education background. Regarding income, seven participants had a middle income, while five participants had a low income. Marital status varied among the participants, with six participants being married with children, two being married without children, and four being single.

All participants had visited China, with two of them having participated in Wushu competitions and five engaging in activities related to Wushu. Additionally, all participants had studied Chinese history, philosophy, and culture, either independently or through various courses. Furthermore, three participants were actively learning the Chinese language.

### Procedure

After participants agreed to take part, the researcher conducted an initial meeting where they signed written consent, asked questions, and scheduled face-to-face interviews. From October to November 2023, all twelve interviews were held in various cafes, lasting one to two hours each and recorded.

The interview questions explored topics such as Chinese culture and martial arts, focusing on participants’ initial interest in Wushu, their experiences and changes over time, their relationships with fellow practitioners, and the impact of training on their self-perception and perceptions of others. Throughout the interviews, the researcher encouraged open dialogue by asking questions and allowing participants to speak freely.

After the interviews, transcripts were reviewed and agreed upon with the participants, with any unwanted details (excluding personal data) removed at their request. Following data analysis, all participants were invited to a debriefing session.

### Ethical considerations

The study design was approved by the director of the Siberian Martial Arts Center and conducted in accordance with the Code of Ethics of the Russian Psychological Society (2006) and the Federal Law of the Russian Federation on Personal Data (2006).

Prior to participating in the study, each participant signed a written consent form, acknowledging their voluntary participation and agreeing to be recorded. They were also informed that they had the right to withdraw from the study at any stage without any negative consequences.

To ensure the confidentiality of the participants, all data collected was anonymized. Any details that could potentially identify individuals were deleted from the transcripts. Additionally, pseudonyms were used for all participants to further protect their identities.

### Data analysis

Thematic analysis was conducted using MAXQDA to extract key themes from the practitioners’ narrative interviews. This analysis followed the methodological guidelines established by Braun and Clarke (2006). The initial phase involved generating codes based on recurring narratives shared by participants, such as their motivations for engaging in Wushu practice. Subsequently, these codes were organized into broader themes; for instance, the theme of “social alienation” emerged from various accounts highlighting the disconnect between Wushu practitioners and those who do not comprehend the significance of their practice.

The final phase involved a rigorous review of the themes to ensure their coherence with the theoretical framework, ultimately identifying connections between the codes and overarching constructs such as “hybrid identity.”

To enhance the robustness of the analysis, triangulation was employed through a systematic coding process. This process was collaboratively conducted by the researcher and two research assistants, thereby strengthening intercoder reliability, as supported by Miles and Huberman (1994). Furthermore, communication with participants within the Wushu center allowed for a direct comparison between participants’ verbal responses and their actual behaviors observed in the training environment. This comparison was used in conjunction with the findings, which were then discussed with the interviewed participants during debriefing sessions. These discussions facilitated critical reflection on the results and contributed to the validation of the analysis.

## Results

Thematic analysis of the interviews revealed a few interconnected themes.

### Theme 1: the formation of a hybrid identity

The first theme is the development of a new hybrid body image. Participants expressed that they pursued Wushu due to their love for martial arts movies and admiration for iconic figures like Bruce Lee, Jackie Chan, Jet Li, and legendary warriors like Ip Man. They aspired to be like these individuals, which motivated them to train in Wushu. However, while they improved their technical skills, they physically remained white Russian males with well-developed muscles. Nevertheless, mentally, they identified themselves as Wushu practitioners and carriers of Chinese culture, inspired by their role models. They referred to this phenomenon as judanren (eggman).^1^

As one participant, S (27 years old, with 2 years of practice), articulated, “On the outside, you are white, but on the inside, you are yellow. Do you understand? I may look like a white guy, but I think like a Chinese master, Shifu.” Another participant, M (53 years old, with 20 years of practice), embraced the concept of judanren, sharing that his practice has facilitated a profound understanding of Chinese culture, including its mentality, philosophy, and history, which has significantly transformed his worldview.

### Theme 2: problems with judanren identity

The hybrid identity of the participants presented several challenges. Firstly, while practicing Wushu, they were expected to uphold its canons and ideals, such as embodying the qualities of a junzi (a noble gentleman). As one participant, L (32 years old, with 2 years of practice), explained, “The standards are very different. In the Russian worldview, you need to have big muscles, be macho, and be able to fight anyone. But in Chinese philosophy, it is better to avoid confrontation. Nobody needs big muscles; you should aim for lean and strong muscles, and it’s not about their size. So, in a shirt, I may look like a skinny nerd, but inside, I am a fierce tiger. It’s a joke, but there is some truth in every joke.”

Consequently, these practitioners often felt like outsiders within the sports and fitness community in Russia, even though some continued to practice in the gym or swimming pool alongside Wushu. Additionally, they faced challenges when visiting China for competitions and training, as they were often perceived as laowais (foreigners) and experienced a sense of rejection from ordinary citizens, despite some being able to speak Chinese.

### Theme 3: uniform as the elimination of contradictions of hybrid identity

The use of accessories and uniforms is crucial in shaping a new identity for practitioners, serving as an extension of their physical selves and helping to reconcile the contradictions inherent in their hybrid identities. Newcomers may begin their journey wearing simple t-shirts, loose trousers, and their own sneakers or gymnastic slippers. However, consistent practitioners are required to adopt proper attire, including a traditional Chinese Wushu yifu uniform and either Feiyue or Budosaga sneakers. These can be purchased directly at the center or ordered from China. The director of the center emphasizes the importance of the uniform in fostering focus and discipline, noting that it not only organizes individuals but also cultivates a sense of unity among practitioners, regardless of race or nationality.

This shared identity helps mitigate feelings of alienation in various social contexts. Participants highlighted the transformative power of the uniform. For instance, Participant P (40 years old, with 10 years of practice) remarked, “When I’m in casual clothes, I feel like a different person compared to when I’m in uniform. People perceive me differently based on my appearance. For example, they might greet me with a namaste gesture, associated with something oriental. The uniform becomes my second skin, distinct from my nationality, skin color, or eye shape.”

The martial arts uniform not only alters how practitioners view themselves but also influences how they are perceived by others. When practitioners wear their uniforms after training, strangers recognize them as Wushu practitioners, transcending national and cultural boundaries. This transformation goes beyond mere physical appearance; it fundamentally shapes the practitioner’s identity and their acknowledgment by others.

Furthermore, the uniform acts as a cultural intermediary, bridging the gap between Russian and Chinese cultures. Participant B (39 years old, with 10 years of practice) explained, “The hybrid identity allows us to build a bridge between Russian and Chinese cultures. After witnessing our performances at fairs or festivals, people approach us to learn about Chinese culture, knowing that we can provide insights not only as cultural translators but as interpreters. This gives us self-confidence, both individually and as a group of practitioners.”

In summary, the uniform plays a vital role in resolving the contradictions of practitioners’ hybrid identities. It unifies them as a social group, transforms their self-perception, and enables them to act as cultural intermediaries between Russian and Chinese cultures.

## Discussion

The thematic analysis of narrative interviews revealed a cultural hybridity termed judanren, referring to individuals who physically present as white Russian males while identifying as Wushu practitioners and custodians of Chinese culture. This bicultural identity departs from traditional studies that primarily focus on immigrants interacting with different cultural groups (Berry, 1980; Bhandari, 2021; Jensen et al., 2011; West et al, 2017). Since Wushu practitioners live within their native environment and represent the dominant culture, their engagement with traditional Chinese culture can be seen as “inner migration,” leading to a unique bicultural identity.

Consequently, the participants in this study assume a bicultural identity, grappling with the tensions between physical appeal and inner self-view. This discord can be resolved through the adoption of a hybrid identity, facilitated by donning a uniform and connecting with like-minded individuals who share an affinity for Chinese traditional culture. As a result, Wushu group members develop a strong collective identity through shared practices and interactions, which reinforce their connection to both Wushu and Chinese culture. This strong sense of group affiliation impacts the formation of participants’ self-concept, as suggested by social identity theory (Tajfel and Turner, 1985).

The emergence of the judanren concept emphasizes the complex interplay between cultural influences and body image perceptions, challenging the Western paradigm of body image as a fixed reference (Landor et al., 2024). This highlights the multifaceted nature of body image formation among Wushu male practitioners in this multicultural context, warranting further investigation.

Acknowledging the non-Western paradigm of body image embraced by these individuals provides a comprehensive understanding of their distinct experiences and the influence of cultural factors on body image. This recognition underscores the significance of incorporating diverse cultural perspectives when investigating body image and its intricate relationship with identity.

In conclusion, the analysis of narrative interviews with Wushu male practitioners at the Siberian Chinese Martial Arts Center reveals a nuanced manifestation of cultural hybridity in body image formation. The judenrein concept is highlighted as illustrating the complex interplay between cultural influences and body image perceptions in a multicultural context.

These findings are seen to extend Social Identity Theory and bicultural identity concepts, particularly in relation to non-Western and hybrid identity formation. Practical implications are offered for instructors in multicultural training environments, emphasizing the necessity of recognizing the diverse cultural backgrounds of their students.

By understanding the unique experiences of these practitioners, the significant role of cultural factors in shaping body image and identity in an increasingly globalized world is appreciated.

## Study limitations

The small sample size limits the generalizability of the findings, as participants were drawn from a single martial arts center in a medium-sized Siberian city. Their experiences may not represent all Wushu practitioners, especially in larger urban areas like Moscow. Future research should include studies in different cities to capture a broader range of experiences and perspectives, considering various genders and cultural contexts.

Additionally, reliance on self-reported data introduces the risk of recall and social desirability biases. To address this limitation, future studies should incorporate observational methods to gain deeper insights into group behaviors and interactions.

Employing mixed-methods approaches can provide a more comprehensive understanding of Wushu practitioners’ experiences. Comparative and longitudinal studies can further explore how these experiences vary across cultural contexts and change over time, enhancing the understanding of hybrid identities within the Wushu community.

## Data Availability

The raw data supporting the conclusions of this article will be made available by the authors, without undue reservation.

## Footnotes

^1^The term “jūdànrén” (鸡蛋人) originated as a Chinese meme that characterizes foreigners in China who genuinely engage with Chinese culture, symbolizing the duality of being perceived as “white-skinned people with a yellow heart” (Babushkina and Kobyakova, 2017, p. 29). Furthermore, this term has been utilized in media studies (Babushkina and Kobyakova, 2017).
